# Feasibility and Safety of Early Cardiac Rehabilitation Using Remote Electrocardiogram Monitoring in Patients with Cardiac Surgery: A Pilot Study

**DOI:** 10.3390/jcm14144887

**Published:** 2025-07-10

**Authors:** Yeon Mi Kim, Bo Ryun Kim, Sung Bom Pyun, Jae Seung Jung, Hee Jung Kim, Ho Sung Son

**Affiliations:** 1 Department of Physical Medicine and Rehabilitation, Korea University Anam Hospital, Seoul 02841, Republic of Korea; 2 Department of Thoracic and Cardiovascular Surgery, Korea University Anam Hospital, Seoul 02841, Republic of Korea; heartistcs@korea.ac.kr (J.S.J.); heejung440@hanmail.net (H.J.K.); hssonmd@korea.ac.kr (H.S.S.)

**Keywords:** cardiac rehabilitation, telerehabilitation, cardiac surgery, exercise test, walk test, ambulatory electrocardiography monitoring

## Abstract

**Purpose**: We aimed to evaluate the safety and feasibility of a remote electrocardiogram (ECG) monitoring-based cardiac rehabilitation (CR) program during an early postoperative period in patients who underwent cardiac surgery. **Methods**: Five days after cardiac surgery, patients were referred to a CR department and participated in a low-intensity inpatient CR program while wearing an ECG monitoring device. Prior to discharge, the patients underwent a cardiopulmonary exercise test (CPET) and squat endurance test to determine the suitable intensity and target heart rate (HR) for home-based CR (HBCR). During 2 weeks of the HBCR period after discharge, patients participated in aerobic and resistance exercises. Electrocardiogram data were transmitted to a cloud, where researchers closely monitored them through a website and provided feedback to the patients via telephone calls. Grip strength (GS), 6 min walk distance (6 MWD), EuroQol-5 dimension (EQ-5D), short-form 36-item health survey (SF-36), and Korean Activity Scale/Index (KASI) were measured at three different time points: 5 d post-surgery (T1), pre-discharge (T2), and 2 weeks after discharge (T3). Squat endurance tests and CPET were performed only at T2 and T3. **Result**: Sixteen patients completed the study, seven (44%) of whom underwent coronary artery bypass graft surgery (CABG). During the study period between T2 and T3, peak VO_2_ improved from 12.39 ± 0.57 to 17.93 ± 1.25 mL/kg/min (*p* < 0.01). The squat endurance test improved from 16.69 ± 2.31 to 21.81 ± 2.31 (*p* < 0.01). In a comparison of values of time points between T1 and T3, the GS improved from 28.30 ± 1.66 to 30.40 ± 1.70 kg (*p* = 0.02) and 6 MWD increased from 249.33 ± 20.92 to 387.02 ± 22.77 m (*p* < 0.01). The EQ-5D and SF-36 improved from 0.59 ± 0.03 to 0.82 ± 0.03 (*p* < 0.01) and from 83.99 ± 3.40 to 122.82 ± 6.06 (*p* < 0.01), and KASI improved from 5.44 ± 0.58 to 26.11 ± 2.70 (*p* < 0.01). In a subgroup analysis, the CABG group demonstrated a greater increase in 6 MWD (102.29 m, *p* < 0.01) than the non-CABG group. At the end of the study, 75% of the patients expressed satisfaction with the early CR program guided by remote ECG monitoring. **Conclusions**: Our findings suggest that early remote ECG monitoring-based CR programs are safe and feasible for patients who have undergone cardiac surgery. Additionally, the program improved aerobic capacity, functional status, and quality of life.

## 1. Introduction

In South Korea, a 25% increase in cardiovascular mortality was observed over the past decade, making it the second leading cause of death in 2022 [1]. Each year, approximately 10,000 patients are admitted to intensive care units following cardiovascular surgery, with a rising trend observed between 2010 and 2019. Aortic surgery has demonstrated the highest mortality rate, followed by coronary artery bypass grafting (CABG) and valve surgery [2].

Cardiac rehabilitation (CR) improves exercise capacity, quality of life, hospital admissions, and mortality rates. Clinical guidelines from Europe, America, and Korea strongly recommend the incorporation of CR for patients with congestive heart failure, valve disease, coronary heart disease, and post open-heart surgery [3,4,5]. Cardiac rehabilitation comprises three phases. Phase 1 begins with early mobilization from intensive care units, aiming to enhance ambulation in the ward. This phase generally involves in-hospital exercises at a CR center with heart rate (HR) and electrocardiogram (ECG) monitoring. Phase 2 represents the early stage of outpatient CR, which typically occurs within 1–3 months after a cardiac operation. In phase 3, patients actively engage in home- and community-based CR exercises and risk factor management, allowing them to sustain lifelong follow-up care in their homes and local communities [6].

Before starting phase 2 CR, typically approximately 4 weeks postoperatively, patients undergo a cardiopulmonary exercise test (CPET). Those categorized as having a moderate-to-high risk of cardiac arrest during exercise are advised to engage in center-based CR (CBCR). However, participation rates in CBCR remain suboptimal, with approximately 30% of eligible patients participating worldwide. This low participation rate can be attributed to challenges such as limited center accessibility, geographical distance, financial constraints, and time constraints [7]. In South Korea, the implementation rate of CBCR stands at only 28%, lower than the global average (12% in secondary medical centers and 41% in tertiary centers), primarily due to a shortage of staff and space [8]. Moreover, following the World Health Organization’s declaration of the pandemic caused by the new coronavirus, SARS-CoV-2, the participation rate in CBCR further decreased [9].

Home-based CR (HBCR) has been introduced as an alternative to CBCR to improve participation. According to a 2019 Cochrane review, HBCR and CBCR demonstrated comparable effects on clinical outcomes and quality of life among individuals with heart failure and acute coronary syndrome [10]. With the improvement in telemedicine, HBCR using remote monitoring devices is increasingly prevalent. By monitoring patients’ objective data, such as HR, ECG, and physical activity, individuals can safely engage in exercise programs, leading to a reduction in anxiety [11].

Studies have found that an early initiation of CR following cardiac surgery not only reduces mortality and shortens hospital stays but also, importantly, does not lead to an increase in adverse events [12]. However, to our knowledge, the majority of previous studies on HBCR with remote monitoring were conducted at least 1 month after surgery or discharge. Consequently, studies exploring the safety and effectiveness of home-based telerehabilitation within the first month following open-heart surgery are lacking.

Therefore, we aimed to evaluate the safety and feasibility of an early CR program using remote ECG monitoring during an early postoperative period in patients who had undergone cardiac surgery.

## 2. Methods

### 2.1. Study Population

This was a single-center prospective study that included patients who underwent open-heart surgery at the Korea University Anam Hospital. The Institutional Review Board of Korea University Anam Hospital approved the study design (approval no: 2021AN0089). Between November 2021 and April 2023, patients who underwent open-heart surgery and were referred to the Rehabilitation Medicine Department for inpatient CR were screened for eligibility.

Patients referred from the Cardiothoracic Surgery Department were screened for eligibility based on predefined inclusion and exclusion criteria. Written informed consent was obtained from all participants prior to enrollment.

Inclusion criteria: aged >18 years; left ventricular ejection fraction (LVEF) > 35%; ability to perform an exercise test; diagnosis of acute coronary syndrome, heart failure, or valvular heart disease and having undergone cardiac surgery; sufficient cognitive ability to understand instructions related to the use of the mobile application and ECG monitoring patch used in the study; and the ability to ambulate independently.

Exclusion criteria: high-risk patients with a history of cardiac arrest or an implantable cardioverter defibrillator (ICD); unstable medical conditions; cognitive impairment interfering with comprehension of the exercise protocol or instructions; musculoskeletal or neurological conditions affecting the lower extremities that would interfere with exercise training; and inability to ambulate independently.

### 2.2. Study Protocol

**Figure 1:**
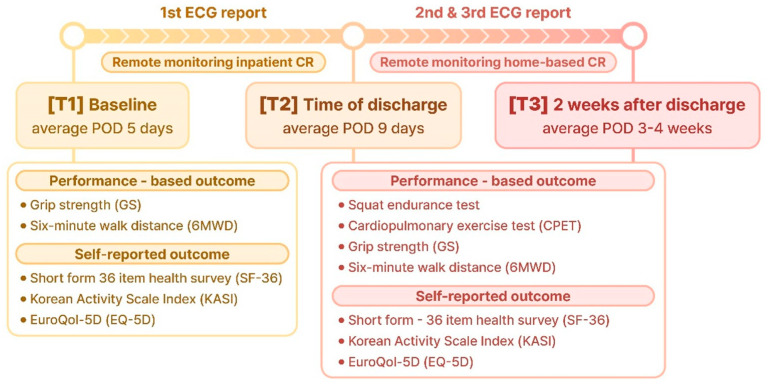
Protocol of the study design.

On average, 5 d postoperatively, the participants initiated a low-intensity inpatient CR program while wearing an ECG monitoring device. Before starting the program, baseline data, including grip strength (GS), 6 min walk distance (6 MWD), the EuroQol-5 dimension (EQ-5D), the short-form 36-item health survey (SF-36), and the Korean Activity Scale/Index (KASI), were collected. Outcomes were measured at three time points: starting CR (average 5 d postoperatively) (T1), before discharge from the hospital (approximately 9 d postoperatively) (T2), and at the end of the study period (approximately 3–4 weeks postoperatively) (T3). Incremental submaximal exercise and squat endurance tests were conducted at two points: T2 and T3 (Figure 1).

Before starting the exercise program, the patients were instructed on proper utilization of the ECG patch monitor and mobile applications. They were advised to wear the patches consistently throughout the study period. The inpatient CR program was conducted five times a week for a total of 1 h. The program consisted of a 10 min warm-up exercise, 10 min of resistance training, 30 min of aerobic exercise, and a 10 min cool-down phase. If the patient experienced dyspnea during the exercise, a brief rest period was encouraged before resuming the program.

Before discharge, approximately 9 d postoperatively, the patients underwent a symptom-limited submaximal exercise test using an ergometer and a squat endurance test. These assessments were aimed at determining the appropriate intensity and target HR for subsequent home-based aerobic and strength exercises.

**Figure 2:**
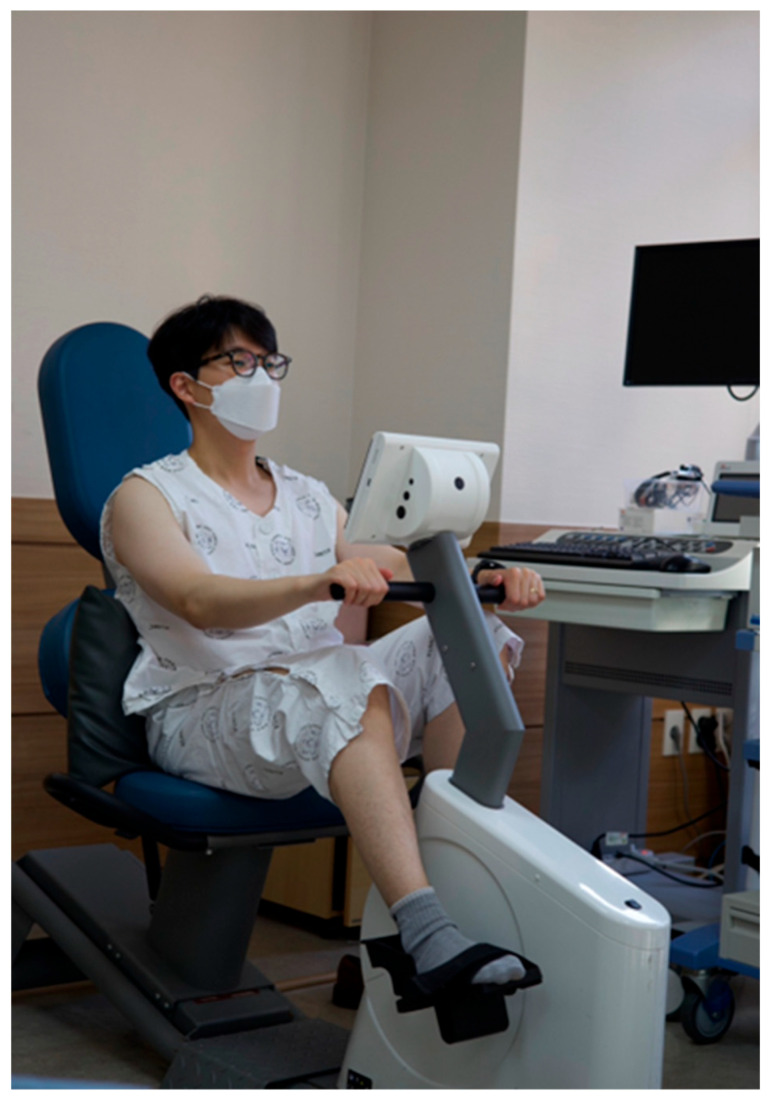
Symptom-limited exercise test with a cycle ergometer (Corival Recumbent cpet 969900; Lode, Groningen, The Netherlands).

For the incremental symptom-limited exercise test, subjects lay on a cycle ergometer (Corival Recumbent cpet 969900, Lode, Groningen, the Netherlands) (Figure 2) and pedaled the bicycle with an initial load of 0 watts and a pedaling frequency of 60 rpm, and the workload was increased by 20 watts every 2 min. The test was terminated if any absolute indications for discontinuation during the examination were observed or if pre-established termination criteria were met (Table 1). During the test, HR, peak oxygen consumption, peak metabolic equivalent of task (MET), maximal work capacity (Wmax), peak respiratory exchange ratio, peak rate of perceived exertion (RPE), peak dyspnea scale, peak angina scale, and total exercise duration were measured. The metabolic equivalent of task was automatically calculated, with one MET defined as the oxygen consumption level during seated rest (equivalent to 3.5 mL of oxygen per kg per min) [13]. The Wmax was determined by the highest intensity that an individual can sustain for 30 s, beyond which it becomes unsustainable. Following 2 weeks of home-based exercise, the patients revisited the hospital for a follow-up exercise test. The test was conducted on a treadmill instead of a cycle ergometer.

A squat endurance test [14] was conducted to assess the intensity of the targeted resistance exercise. The participants leaned against the wall, stood with their feet shoulder-width apart, and initiated squats by flexing their knees at a 90° angle from a standing position. The test was performed with the intensity set to 40–60% of the HR reserve, determined through symptom-limited maximal exercise testing, and continued until it reached a slightly challenging level (RPE 13).

After discharge, the patients underwent a 2-week HBCR program. For aerobic exercises, the patients were informed of their target HR using the Karvonen formula [15], with an exercise intensity set at 40–55% of the HR reserve, determined from an incremental submaximal exercise test. The target HR can be estimated as follows: (target HR = resting HR + [0.40 − 0.55] × [peak HR − resting HR]). Patients were also advised to stop exercising when they reached RPE 13. For anaerobic exercise, since all the patients maintained their HRs within the target range (40–60% of peak HR) when reaching RPE 13, we recommended that they perform the same number of squat repetitions for their home exercise.

### 2.3. Remote ECG Monitoring System

**Figure 3:**
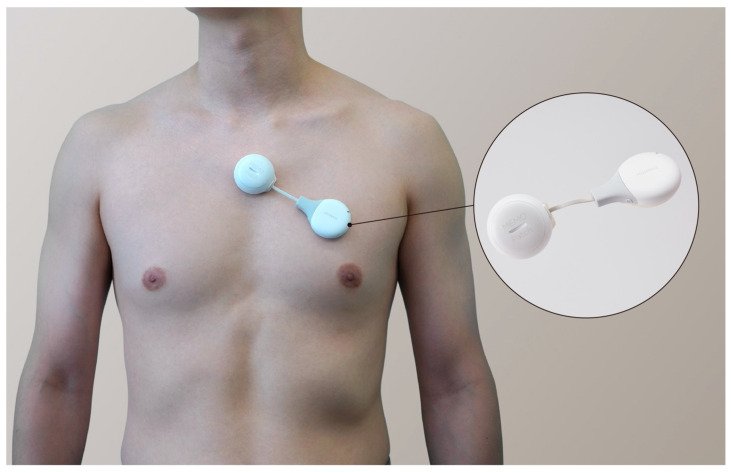
MEMO Patch and attachment sites.

To monitor remote ECG data, we used the MEMO Patch (MEMO Patch, HUINNO Co., Ltd., Seoul, South Korea) (Figure 3), the first ambulatory ECG device approved by the Korean Food and Drug Administration. This adhesive single-lead patch can store ECG data for up to 14 d. A cohort study in Korea showed that using the patch for 7 d of continuous ECG monitoring was more effective in detecting supraventricular tachycardia than the traditional 24 h Holter monitor [16]. The recorded ECG data from the patch were subjected to analysis using MEMO artificial intelligence (AI), which was constructed and trained by HUINNO for arrhythmia classification [17]. The AI algorithm employed in this study integrates multiple components tailored for specific tasks within cardiac signal analysis. First, it uses a convolutional neural network architecture with skip connections engineered specifically to detect episodic arrhythmias and filter out noise signals, enhancing the reliability of detection. Second, a dedicated segmentation and classification network is implemented, which is finely tuned to identify and delineate the P, QRS, and T segments of the ECG, enabling detailed feature extraction from these critical signal components. Finally, a sophisticated postprocessing algorithm synthesizes the information from the previous stages to refine the overall analysis. This multifaceted approach ensures a robust and precise initial analysis by leveraging deep learning techniques to improve diagnostic accuracy.

After the ECG data were analyzed using AI, the technicians manually reviewed and verified the AI-generated diagnosis. If necessary, the diagnosis was revised after confirmation by cardiologists.

To protect data privacy, only a randomly generated test ID and the deidentified ECG signal for each test were transmitted to the cloud platform. In addition, the ECG signals were encrypted using the AES (Advanced Encryption Standard) algorithm during network transmission to ensure security. Patient information is stored separately from the ECG data in an isolated database along with the corresponding test ID. This information was not used during the transmission process and is accessible only to authorized medical personnel.

**Figure 4:**
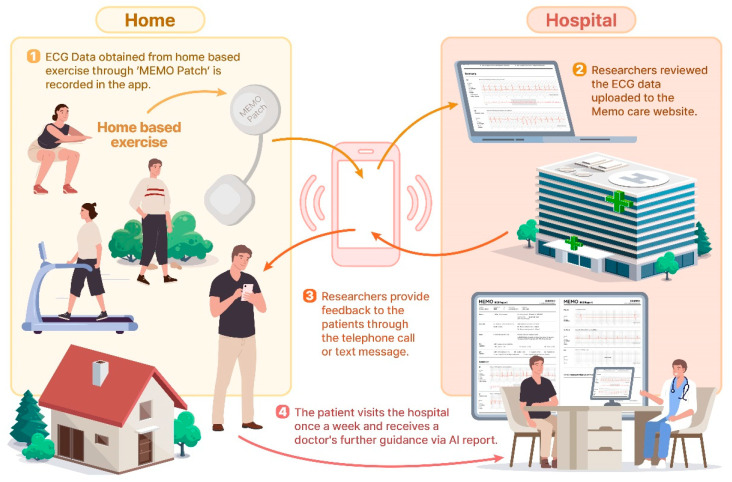
Overview of rehabilitation programs using remote monitoring devices.

The patients were instructed to wear the patch consistently throughout the study period. In addition, they were provided with mobile phones featuring internet connectivity, and the device was preloaded with an application connected to the ECG patch. During the daily exercise, the patients activated the application to transmit their ECG data, and upon completion, they deactivated the application. Subsequently, the ECG data were transmitted in real time to the HUINNO Cloud. Medical doctors closely monitored the data using the MEMO Care website and provided feedback to the patients through telephone calls (Figure 4). If ventricular fibrillation, sustained ventricular tachycardia, or ST segment elevation > 1 mm was observed in the ECG data, the researchers planned to recommend discontinuing home-based exercises and scheduling an earlier hospital visit via telephone calls. In such cases, the researchers also intended to consult with a cardiologist and transition the patient to 100% onsite rehabilitation. Additionally, the patients were instructed to report “patient-triggered events” by pressing the button on the patch if they experienced angina chest pain, dizziness, or syncope.

**Figure 5:**
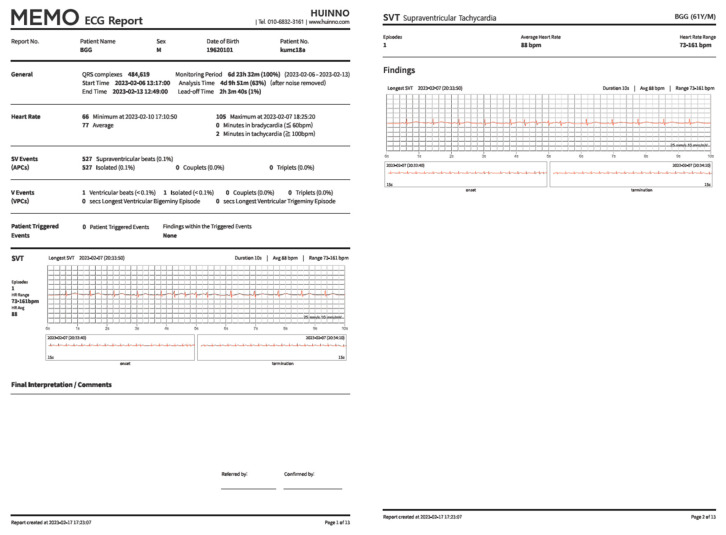
Example of weekly artificial intelligence-generated report.

At the end of each week, the participants visited the hospital to return the patch, where doctors offered further guidance by reviewing the weekly reports available on the website. The weekly report covers the entire monitoring period and includes data on minimum, average, and maximum HRs, as well as details on the number and duration of various arrhythmia events, such as atrial fibrillation (AF), supraventricular tachycardia, ventricular fibrillation, atrial premature complexes, ventricular premature complexes, and patient-triggered events (Figure 5).

### 2.4. Physical Assessments

#### 2.4.1. GS

The GS test is a widely employed method for assessing muscle strength and serves as a predictor of various health outcomes, including cardiovascular mortality, length of hospital stay, functional status, and perioperative complications [18]. To assess GS, the participants were instructed to apply maximal force alternately with their left and right hands on a handheld dynamometer (JAMAR PLUS + Digital Hand Dynamometer; Sammons Preston Rolyan, Bolingbrook, IL, USA) and repeat this process twice. Following two attempts, maximal grip power (kg) was recorded. The greater GS between the two hands was used for further analysis.

#### 2.4.2. 6 MWD

The 6 MWD is a simple and safe test that has been found to significantly reflect functional status and activities of daily living [19]. The participants were instructed to walk along a 30 m corridor for 6 min, aiming to cover as much distance as possible while maintaining a perceived intensity between 3 (moderate) and 4 (somewhat strong) on the Borg CR scale (CR 10) [20]. Rest was permitted if participants felt exhausted, and the test was halted if they experienced dyspnea or chest pain that impeded daily activities. Elapsed time was recorded for the patients at each minute of assessment without additional feedback or encouragement. Oxygen saturation, blood pressure, and HR were measured before and after the test, and the distance covered during the 6 min period was recorded.

### 2.5. Self-Reported Survey

#### 2.5.1. SF-36

The SF-36 is a widely used self-reported quality-of-life questionnaire consisting of 36 items. It assesses physical health using physical function, physical role, bodily pain, and general health. Additionally, it evaluates mental health using vitality, social function, role emotional, and mental health. These eight subscales were further divided into 35 items, with the remaining one addressing health change perception, for a total of 36 detailed items. Assigning scores to the detailed items within each of the eight subscales and adding them yields a total score ranging from 0 (indicating the poorest health) to 100 (indicating the best health). The scores can be summarized into two main areas: the physical component summary (PCS) and the mental component summary (MCS). In the general population, scores for each component are expected to have a mean of 50 and a standard deviation of 10 [21]. We used the Korean version of the questionnaires to ensure that patients can easily comprehend and complete the test [22].

#### 2.5.2. KASI

The Korean Activity Scale/Index (KASI) [23] is a Korean-translated version of the Duke Activity Status Index, which is a 12-item scale survey designed to assess functional capacity and quality of life. This test demonstrated a significant correlation between peak oxygen uptake [24] and the occurrence of major adverse cardiac events [25]. The participants were asked to self-assess the questionnaires, and the total scores were determined based on their responses.

#### 2.5.3. EQ-5D

The EQ-5D was used to assess health-related quality of life in our study [26]. It consists of five dimensions concerning current health status (mobility, self-care, usual activities, pain/discomfort, and anxiety/depression), with each dimension consisting of a three-point scale (no problem, moderate problem, and severe problems). Responses to each question were transformed into final scores using Korean value set calculations, ranging between −0.171 and 1. A score of 1 indicates a “healthy state without problems,” while a score of 0 represents “death,” and scores below 0 indicate a state “worse than death.” [27].

#### 2.5.4. Satisfaction Questionnaire on Remote ECG Monitoring

At the end of the study period (T3 time point), participants completed a self-administered questionnaire to assess their satisfaction with the remote CR program utilizing the ECG patch. Satisfaction was measured using a 5-point Likert scale, ranging from 1 (very dissatisfied) to 5 (very satisfied). For analysis, each score was converted to a 20-point increment, yielding a total possible score of 100. The mean score was then calculated to determine overall satisfaction.

### 2.6. Statistical Analysis

Statistical analyses were performed using IBM SPSS Statistics for Windows version 26. To compare baseline characteristics between CABG and non-CABG groups, the Student’s t-test was used for continuous variables, while the chi-square test was used for categorical variables. A mixed linear model was used to analyze changes in repeatedly measured primary and secondary outcomes over time. The model incorporated covariates such as time, group, 6 MWD, EQ5D, SF36, KASI, squat endurance test, and measurements obtained using submaximal exercise tests. There were no missing values, and the study data met the assumption of normality. The variables (GS, 6 MWD, squat endurance test) were fitted to the model under an AR (1) covariance structure, while the variables (KASI, EQ5D, SF36, peak VO_2_, peak HR) were fitted under an unstructured (UN) covariance structure. The variables (peak MET, total exercise duration) were fitted to the model under an CS (compound symmetry) covariance structure. Model selection was based on the Akaike Information Criterion (AIC), with the model exhibiting the lowest AIC value chosen as the optimal model. Statistical significance was considered at *p* < 0.05.

## 3. Results

### 3.1. Baseline Characteristics

**Figure 6:**
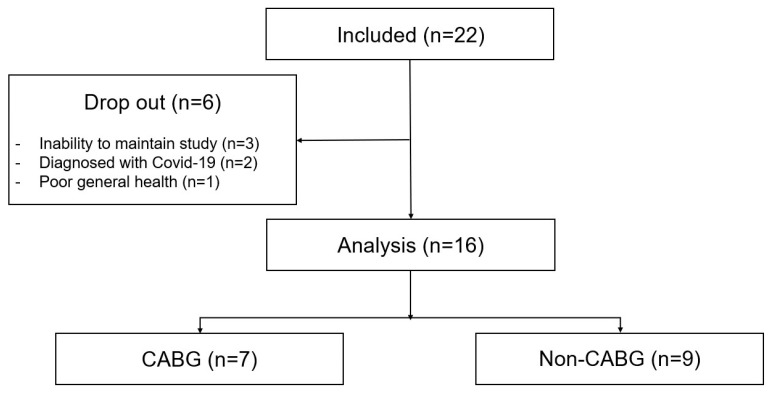
Participant flow diagram.

Baseline demographics and disease-related characteristics of the participants are summarized in Table 2. Of the 22 patients enrolled in the study, six dropped out during the follow-up period. Three patients withdrew from the study due to an inability to maintain continuous observation, as they did not routinely wear the monitoring device or adhere to the study protocol. Two patients were excluded because they were diagnosed with COVID-19. One patient discontinued participation due to poor general health (Figure 6). Sixteen patients, with an average age (standard deviation) of 63.38 (1.89) years, successfully completed the study, and twelve (75.0%) of them were males. Median (interquartile range [IQR]) time to the initiation of CR was 5 (5–6) d. Among the participants, seven (43.8%) had undergone CABG, seven (43.8%) had undergone valve replacement surgery, one had undergone total arch replacement surgery (6.3%), and one had received coronary artery fistulectomy (6.3%).

Comparison between patients who had undergone CABG and those who had undergone non-CABG surgery showed no significant differences (*p* > 0.05).

### 3.2. Changes over Time in Participant Outcomes

Comparison between the second assessment at T2 and the final assessment at T3 (T2–T3) showed that peak VO_2_ increased from 12.39 ± 0.57 mL/kg/min to 17.93 ± 1.25 mL/kg/min (*β* = 6.2, 95% CI: 3.6 to 7.4, *p* < 0.01), peak MET improved from 3.54 ± 0.28 mL/kg/min to 5.11 ± 0.28 mL/kg/min (*β* = 6.1, 95% CI: 1.0 to 2.1, *p* < 0.01), and peak HR increased from 110.19 ± 5.53 to 122.12 ± 5.53 (*β* = 2.5, 95% CI: 1.6 to 22.3, *p* < 0.01). Additionally, the squat endurance test demonstrated improvement from 16.69 ± 2.31 to 21.81 ± 2.31 (*β* = 5.5, 95% CI: 3.1 to 7.1, *p* < 0.01).

**Figure 7:**
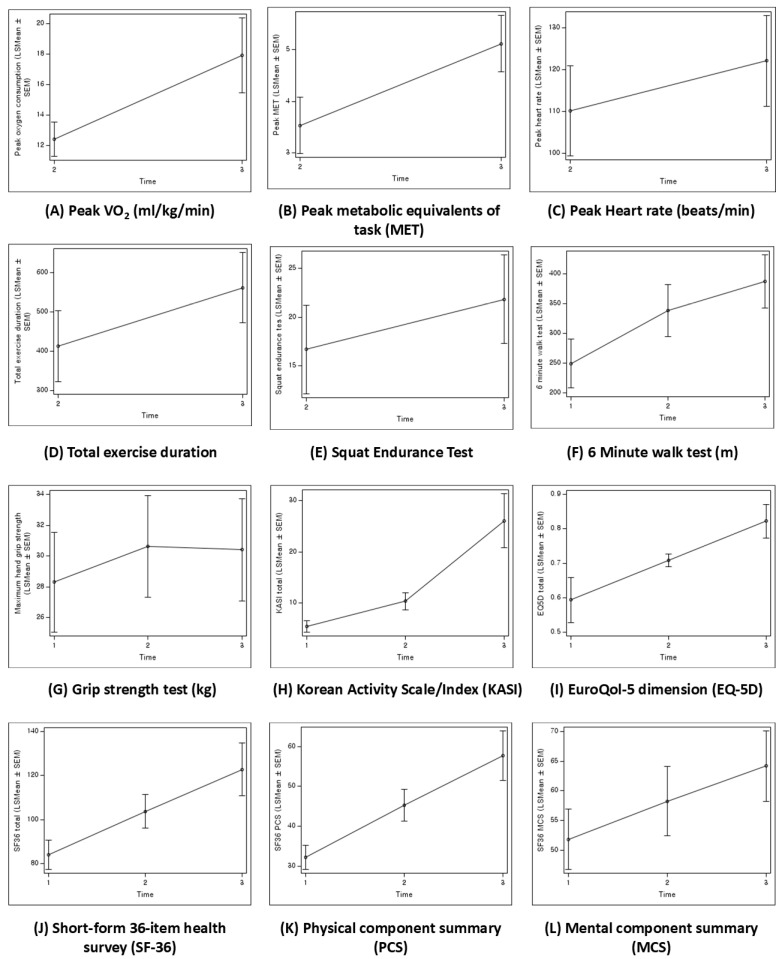
Changes in peak VO_2_ (**A**), peak MET (**B**), peak HR (**C**), total exercise duration (**D**), squat endurance test (**E**), 6 MWD (**F**), grip strength (**G**), KASI (**H**), EQ-5D (**I**), and SF-36 (**J**–**L**). HR, heart rate; MET, metabolic equivalent of task; 6 MWD, 6 min walk distance; EQ5D, EuroQol-5 dimension; SF, short-form 36-item health survey; KASI, Korean Activity Scale/Index.

Compared to the initial assessment at T1, the GS showed improvement from 28.30 ± 1.66 kg to 30.40 ± 1.70 kg at T3 (*β* = 2.8, 95% CI: 0.2 to 4.0, *p* = 0.02). However, no significant change was noted between T2 (30.62 ± 1.68 kg) and T3. The 6 MWD increased from 249.33 ± 20.92 m at T1 to 387.02 ± 22.77 m at T3 (*β* = 6.9, 95% CI: 88.6 to 186.8, *p* < 0.01). The EQ5D scores improved from 0.59 ± 0.03 at T1 to 0.82 ± 0.03 at T3 (*β* = 8.5, 95% CI: 0.2 to 0.3, *p* < 0.01). The KASI improved from 5.44 ± 0.58 at T1 to 26.11 ± 2.70 at T3 (*β* = 7.4, 95% CI: 13.7 to 27.7, *p* < 0.01). Total scores of the SF-36 showed improvement from 83.99 ± 3.40 at T1 to 122.82 ± 6.06 at T3 (*β* = 5.9, 95% CI: 22.2 to 55.5, *p* < 0.01). The PCS of SF-36 significantly improved from 32.16 ± 1.55 at T1 to 57.69 ± 3.20 at T3 (*β* = 9.0, 95% CI: 18.4 to 32.7, *p* < 0.01). Although the MCS score showed a significant increase (*β* = 3.3, 95% CI: 3.1 to 21.6, *p* < 0.01) between T1 (51.83 ± 2.59) and T3 (64.15 ± 3.02), no significant difference was noted between T2 and T3 (*p* = 0.09) (Figure 7).

In the subgroup analysis, the CABG group demonstrated a greater increase in 6 MWD (102.29 m, *p* < 0.01) than the non-CABG group. However, no significant differences were observed in other measurements between the two groups.

### 3.3. ECG Recording Results

Over the course of the study period, the average ECG monitoring duration increased from 125 h to 179 h. The median occurrence of atrial premature complex changed from 261 to 155, while that of ventricular premature complex decreased from 135 to 88.5. Atrial fibrillation was reported in five patients, and non-sustained ventricular tachycardia events were reported in six patients.

### 3.4. Adverse Effects

Regarding safety concerns, there were no reported serious adverse events, such as hospitalization, cardiac arrest, or death. Throughout the study period, no instances of sustained ventricular tachycardia or fibrillation were recorded using an ECG monitoring device.

### 3.5. Patient Satisfaction Regarding the Remote Monitoring-Based HBCR Program

Based on the final self-report questionnaires, 12 (75.0%) patients completed the HBCR program with satisfactory remote ECG monitoring. Participants primarily expressed satisfaction because they felt reassured by the real-time HR monitoring through the mobile application (75.0%) and because they were able to review the automatically saved ECG records later (68.8%). Additionally, they appreciated the ability to regulate exercise intensity with alarms set above individual HR thresholds. In terms of areas that needed improvement, four (25.0%) patients reported experiencing a skin rash and itching sensation at the patch attachment site. Three (18.8%) patients mentioned that the patch detached easily when sweating occurred after the exercise. Two (12.5%) patients reported Bluetooth disconnection. Additionally, three (18.8%) patients, with an average age of 73.3 years, encountered difficulties in manipulating the mobile application due to their advanced age.

## 4. Discussion

The principal finding of this study was that initiating early CR within 1 month after first-time open-heart surgery, using an ECG monitoring device, resulted in significant improvements in exercise capacity, quality of life, and various functional outcomes.

To the best of our knowledge, the present study is the first to validate the safety and effectiveness of initiating early rehabilitation using a remote ECG device coupled with real-time feedback among patients undergoing different cardiac surgeries, such as CABG, valve operations, and aortic surgeries. Previous studies using ECG monitoring systems for remote post open-heart surgery monitoring primarily began exercise programs during the phase II period [28,29], with a lack of studies initiating home exercise programs within 2 weeks postoperatively [30]. Even though all patients who undergo open-heart surgery are recommended to receive CR, the CR participation rate remains suboptimal [8]. Home-based exercise training with a remote ECG monitoring system could serve as an effective alternative model to achieve this objective. Previous studies have indicated that telerehabilitation with various monitoring systems has a comparable effect to hospital-based rehabilitation [31,32]. Recent CR guidelines advocate for ECG-monitored exercise during an early post-onset period [5,33]. Early initiation of CR following cardiac surgery is known to shorten hospital stays [12]. As early discharge has the potential to reduce costs and improve patient satisfaction, and has been shown to be safe, it is becoming a growing trend in the management of cardiovascular conditions [34]. Initiating ECG-monitored exercise during the early period aligns with this trend and may help patients achieve early discharge. An ECG monitoring system, capable of recording one-lead cardiac rhythm, detecting arrhythmias, and monitoring HR, can enhance adherence to CR. In our study, we observed a notable increase in the total duration of patients wearing the ECG-monitoring device over a span of approximately 3 weeks. Additionally, receiving real-time feedback from the medical team instills confidence and a sense of safety among patients, thereby further improving their participation rate.

Although our study only included an intervention group without a control group that did not participate in the exercise program, our results showed improvements in various exercise parameters (peak VO_2_, MET, and peak HR) during incremental exercise tests after 2 weeks of home-based exercise programs. These findings support previous reports highlighting that telerehabilitation offers benefits comparable to standard rehabilitation therapy [11,35]. Additionally, the total KASI and EQ-5D scores, which indicate the patient’s activity levels and quality of life, showed a significant increase compared to the initial assessment and discharge. As KASI scores are known to correlate with oxygen capacity [24], this suggests that the program plays a role in enhancing cardiovascular function. Similarly, the total SF-36 score significantly increased after 2 weeks of CR. The PCS measures of the SF-36 demonstrated significant improvement in the final test compared with the initial assessment and discharge status. However, only the MCS measures of SF-36 showed a significant change between the final and initial measurements. No significant differences were observed between the final follow-up and discharge statuses. These improvements signify that despite the burden of open-heart surgery, the participants demonstrated significant enhancements in physical function and overall quality of life after completing the 2-week exercise program.

From a safety perspective, conducting HBCR with remote monitoring shortly after open-heart surgery, within 2 weeks post operation, did not increase the risk of postoperative adverse events. Atrial fibrillation is the most common complication after cardiac surgery, occurring in 15.0–40.0% of cases after CABG and 37.0–50.0% after valve surgery [36]. Non-sustained ventricular arrhythmias are reported in up to 36.0% of cases [37], whereas sustained ventricular arrhythmias occur in 0.4–1.4% of cases after cardiac surgery [38]. In our study, AF was detected in five (31.3%) patients, while non-sustained ventricular tachycardia was observed in six (37.5%). Among them, four patients experienced AF for the first time postoperatively during a remote ECG monitoring exercise training session. However, the duration of AF in these cases was brief, ranging between 10 and 6 min. Throughout the training session, none of the participants experienced life-threatening events, such as sustained ventricular tachycardia or ventricular fibrillation. Furthermore, no instances of patients being readmitted to the hospital or emergency department were noted during the program. Ennis et al. observed that commencing exercise training as early as 2 weeks after sternotomy enhances functional outcomes and the 6 MWD sooner [39]. This early intervention allows patients to regain social function and economic productivity earlier, contributing to an improved quality of life without any substantial additional risks. Consequently, the patients were able to achieve aerobic exercise capacity sooner, potentially leading to earlier improvements in functional activity. However, in Korea, exercise therapy is still postponed for 4 weeks owing to general condition and suture site concerns following cardiac surgery [5,40]. Our findings suggest that initiating remote ECG monitoring-based HBCR within 2 weeks postoperatively can be safely implemented in patients who have recently undergone cardiac surgery.

Through feedback gathered from patient surveys, we identified key areas for enhancing the effectiveness of remote CR. A smartphone application integrated with an ECG monitoring system has great potential for HBCR programs [41]. However, to encourage broader usage, ensuring that patients can easily use both the application and the device is crucial. In our study, 18.8% of the participants reported difficulties with the mobile application. Given that many cardiac surgery patients are older and less familiar with mobile technology, simplifying procedures such as device application and removal, as well as app navigation, is essential. Additionally, 12.5% of patients faced challenges with Bluetooth connectivity, emphasizing the need to improve internet stability for broader and safer adoption of telerehabilitation. Moreover, addressing skin issues is crucial, as participants cannot readily access advice or patch-exchange services while engaging in home-based exercises. Although these issues did not lead to reduced adherence, improving patches is essential for alleviating attachment problems and skin irritation.

Regarding the psychological effects of remote ECG monitoring, home-based CR has been shown to alleviate symptoms of depression and anxiety compared to usual care [11]. Additionally, Xizen et al. reported that monitoring vital signs during home-based rehabilitation can help reduce exercise-related fear [42]. The significant improvement in the MCS score of the SF-36 observed in our study from baseline to final measurements supports these prior findings. However, as comprehensive investigations into the psychological impact of CR involving remote ECG monitoring remain scarce, further research in this area is warranted. Moreover, individualized education that addresses cognitive and behavioral barriers has been shown to enhance patient adherence to exercise programs [43]. Thus, future studies should explore whether tailoring education and exercise regimens based on patients’ psychological profiles can improve both adherence and psychological outcomes in the context of home-based CR.

### Limitation

This study has several important limitations. First, the absence of a control group that did not receive exercise training made it challenging to accurately gauge the true effect of CR on functional outcomes and aerobic capacity. Second, the majority of the participants were males aged 50–70 years, and because of the small sample size, generalizing the study findings is difficult. Third, the initial submaximal exercise test used a cycle ergometer to minimize the burden on patients, whereas the follow-up test employed a treadmill. This discrepancy may have introduced heterogeneity into the final measurements. Fourth, since the final measurements were conducted about 3–4 weeks postoperatively, there were no long-term follow-up results regarding functional outcomes or aerobic capacity. In the future, a large-scale randomized controlled trial with long-term follow-up is warranted to validate the findings of our study.

## 5. Conclusions

The findings of this study suggest that starting HBCR exercise training within 1 month after cardiac surgery, combined with remote ECG monitoring, leads to beneficial outcomes for aerobic capacity, functional status, and quality of life. No significant adverse events were reported during the study period. These results provide valuable insights for clinicians, enabling them to confidently design HBCR with ECG monitoring within the first month after an open-heart surgery. Further studies on CR programs using various wearable devices capable of tracking diverse physical activities of patients, including ECG monitoring, is necessary for continued advancement.

## Figures and Tables

**Table 1:** Termination criteria for the symptom-limited submaximal exercise tests.

Absolute Indication for Stop Test	Pre-Established Termination Criteria
- ST-segment elevation > 1 mm without abnormal Q waves in the ECG channels, excluding V1 and aVR - Decrease in SBP > 10 mmHg or a drop below resting SBP - Severe or higher-grade angina (angina scale grade 3–4) - Able to ambulate without physical assistance - Exacerbation of neurological symptoms (e.g., dizziness and ataxia) - Cyanosis or pallor - Persistent ventricular tachycardia - Patient’s desire to discontinue the test	- HR > 120 beats per minute - Reaching 70% of the maximum predicted heart rate based on the patient’s age - Achieving a predetermined MET level (typically 5–7) - Uncontrolled medical condition - RPE (rate of perceived exertion) exceeding 15 on the Borg 6–20 grade scale

ECG = electrocardiogram, SBP = systolic blood pressure, HR = heart rate, MET = metabolic equivalent of task, RPE = rate of perceived exertion.

**Table 2:** Baseline clinical characteristics and initial 6 MWD, EQ5D, KASI, and SF36 results (*N* = 16).

Variables		Subgroup Analysis		
	Total (*N* = 16)	CABG (*N* = 7)	Non-CABG (*N* = 9)	*p*-Value
Age (years)	63.38 ± 1.89	64.43 ± 7.35	62.56 ± 8.03	0.639
Sex, male/female	12 (75)/4 (25)	12 (86)/1 (14)	6 (67)/3 (33)	0.536
Height (cm)	164.63 ± 2.19	164.33 ± 8.01	166.63 ± 9.62	0.618
Weight (kg)	66.23 ± 2.40	67.71 ± 6.56	65.08 ± 11.73	0.604
Body mass index (kg/m^2^)	24.05 ± 0.56	25.04 ± 1.31	23.28 ± 2.54	0.096
Time to start cardiac rehabilitation (days)	5.00 (5.00; 6.00)	5.00 (4.00; 6.00)	6.00 (5.00; 6.00)	0.174
Grip strength test (kg)	28.19 ± 1.82	28.24 ± 6.83	28.04 ± 8.03	0.959
6 min walk distance (m)	262.00 ± 21.04	237.57 ± 43.06	281.00 ± 104.69	0.283
Korean Activity Scale/Index (KASI)	4.90 (3.70; 7.08)	4.90 (3.70; 4.90)	6.70 (3.70; 9.70)	0.174
EuroQol-5 dimension (EQ5D)	0.68 (0.51; 0.68)	0.68 (0.47; 0.68)	0.68 (0.51; 0.70)	0.758
Short-form 36-item health survey (SF36)				
Total score	86.79 ± 4.14	84.82 ± 9.08	88.33 ± 21.12	0.689
Physical component summary (PCS)	33.61 ± 1.43	34.20 ± 3.55	33.16 ± 7.20	0.733
Mental component summary (MCS)	53.18 ± 3.26	50.63 ± 6.74	55.17 ± 16.56	0.471

Values represent mean ± standard deviation, median (interquartile range), or number (%) of cases. CABG = coronary artery bypass grafting.

## Data Availability

This study was registered with the Clinical Research Information Service (CRIS) under trial registration number KCT0006444 on 13 August 2021.

## Abbreviations

CABG	coronary artery bypass grafting
CR	cardiac rehabilitation
HR	heart rate
ECG	electrocardiogram
CPET	cardiopulmonary exercise test
CBCR	center-based cardiac rehabilitation
HBCR	home-based cardiac rehabilitation
GS	grip strength
6 MWD	6 min walk distance
EQ-5D	EuroQol-5 dimension
SF-36	short-form 36-item health survey
PCS	physical component summary
MCS	mental component summary
KASI	Korean Activity Scale/Index
MET	metabolic equivalent of task
Wmax	maximal work capacity
RPE	rate of perceived exertion
AI	artificial intelligence
AF	atrial fibrillation
